# External Workload Compared Between Competitive and Non-Competitive Matches for Professional Male Soccer Players

**DOI:** 10.2478/hukin-2022-0057

**Published:** 2022-09-08

**Authors:** Jose Asian-Clemente, Bermardo Requena, Adam Owen, Alfredo Santalla

**Affiliations:** 1 Department of Sport sciences, Universidad Pablo de Olavide, Sevilla, España; 2 Football Science Institute, Granada, España; 3 Univ Lyon, LIBM EA7424, University Claude Bernard Lyon 1, Villeurbanne, France; 4 Hebei China Fortune Football Club, Langfang Stadium, Hebei Province, China

**Keywords:** soccer, training, small-sided games, GPS, time-motion analyses

## Abstract

The purpose of this study was to compare the external load in competitive (official) and non-competitive matches (friendly, training and modified-sided games) in professional soccer players. Time-motion data for 10 elite male soccer players (age = 20.1 ± 2.1 years; body height = 178.8 ± 5.9; body mass = 71.4 ± 7.3; % body fat = 11.0 ± 1.1 and VO_2max_ = 55.96 ± 3.3) from a professional Spanish first division team were recorded during official (n = 12), friendly (n = 7) and training (n = 6) matches and a 5 vs. 5 + goalkeepers modified-sided game (n = 3). GPS devices were used to monitor players’ external loads: total distance covered, distance covered at different speeds (<13.9 km·h^-1^, >14, >18, >21 and >25 km·h^-1^), peak speed (km·h^-1^), and the number of accelerations and decelerations (1.5–2.5 m·s^-2^, 2.5–4 m·s^-2^ and 4–8 m·s^-2^). One-way analysis of variance of the magnitude-based inference was used to determine differences between matches. Data indicated that official matches scored statistically higher peak speeds (ES = 1.40–2.20). In modified-sided games more total distance was covered at <13.9 km·h^-1^ and >14 km·h^-1^ than in regular matches (ES = 0.72–2.21), but lower distances were covered at >21 km·h^-1^ and >25 km·h^-1^ than in official and friendly matches (ES = 0.51–2.53) and at >25 km·h^-1^ than in training matches (ES = 0.92). Likewise, the modified-side games showed a greater number of accelerations and decelerations than other types of matches (ES = 1.46–2.51). This work shows that friendly and training matches, in conjunction with modified-side games, are suitable tools to prepare soccer players for official matches.

## Introduction

Despite the worldwide popularity of soccer, many uncertainties concerning the game’s multidimensional requirements and consequently uncertainties when planning for teams’ optimal training and conditioning processes still exist (Abrantes et al., 2012). Throughout the last decade, a substantial growth in research related to specific training methods in soccer has been observed, with a strong emphasis on the effects of small-sided games (SSGs) (Aguiar et al., 2012; Halouani et al., 2017; Owen et al., 2020; Sarmento et al., 2018). SSGs are modified games played on reduced pitch areas, often using adapted rules, and involving a smaller number of players than full-size soccer matches (Hill-Haas et al., 2011). For the technical and conditioning staff associated with soccer, SSGs are fundamental to the simultaneous development of technical and tactical skills under greater than normal specific physical loads (Hill-Haas et al., 2011). Recent literature has also highlighted that SSGs maintain higher levels of players’ motivation in unpredictable training environments (Badin et al., 2016; Halouani et al., 2017; Selmi et al., 2018a, 2018b).

Comparisons of running activity between competitive matches and different SSG types are necessary for coaches and practitioners in professional soccer (Clemente et al., 2019b). These comparisons are suggested to allow specific drills that elicit similar, greater or lower loads compared to actual match-play, with the aim of providing an optimum training intensity stimulus (Owen et al., 2014). However, very few studies have compared the running demands of competition and SSGs with the intention of achieving more specific adaptations and better performance in soccer players (Gómez-Carmona et al., 2018). To our knowledge, only two studies have compared SSG demands with friendly competitions (Casamichana et al., 2012; Dellal et al., 2012). However, the data obtained were inconclusive because one study found that during SSGs more running activity involving sprints and high intensity was performed (Dellal et al., 2012), while the other described a greater amount of sprinting, with more duration and distance, in friendly matches (Casamichana et al., 2012). There have also been few studies comparing the running activity in these tasks with official matches. These studies indicate that SSGs can produce overtraining in acceleration and deceleration demands, but underestimate high intensity running compared to official matches for professional (Clemente et al., 2019b; Dalen et al., 2019; Martin-Garcia et al., 2019) and semiprofessional players (Gómez-Carmona et al., 2018). In all cases, studies have used SSGs with lower relative areas per player than those used in official matches (<300 m^2^). Since the area of play is one of the most influential factors in the running activity of soccer players (Castellano and Casamichana, 2013; Clemente et al., 2019a; Gaudino et al., 2014; Lacome et al., 2018), it is necessary to determine running demands in modified-sided games (MSGs) in terms of the relative area per player compared to official soccer matches. Similar to SSGs, training matches are a very popular means used by coaches in training sessions because it is thought that they replicate the technical-tactical requirements and running activity of competitive matches (Martin-Garcia et al., 2019). However, running demands of these types of matches have never been compared with friendly and official matches. Therefore, the purpose of this study was to compare the external loads in competitive (official) and noncompetitive matches (friendly, training and MSG) in professional soccer players.

## Methods

### Participants

Ten male professional soccer players participated in this study. The participants’ characteristics are presented in Table 1. Athletes belonged to the reserve squad of a Spanish La Liga club. All of them received a clear explanation of the study, including the potential risks and benefits of participation, and written consent was obtained. The experimental protocol was approved by the local Institutional Ethics Committee of the University of Pablo de Olavide according to the principles outlined in the Declaration of Helsinki.

**Table 1:** Participants’ characteristics.

Age (years)	Body height (cm)	Body mass (kg)	% body fat (Faulkner)	VO_2max_ (mL^-1^ · kg^-1^ · min^-1^)
20.1 ± 2.1	178.8 ± 5.9	71.4 ± 7.3	11.0 ± 1.1	55.96 ± 3.3

*Data are mean ± SD. Abbreviations: VO2max **=** maximal oxygen uptake*.

### Material and Instruments

Data were collected using portable GPS devices (SPI Pro X; GPSports Systems, Canberra, Australia) operating at a sampling frequency of 5 Hz and an accelerometer operating at 100 Hz. Players wore a special harness that enabled these devices to be fitted to the upper part of their backs. The GPS device was activated 15 minutes before kick-off, in accordance with the manufacturer’s instructions. After recording, the data were downloaded to a PC and analyzed using a software package (SPI Pro X; GPSports Systems, Canberra, Australia). Players were familiarized with the use of these devices and the SSG format used, prior to the commencement of the study. The validity and reliability of the GPS system have been previously reported and widely used with soccer players (Casamichana et al., 2012; Castellano et al., 2011).

### Variables

**Figure 1:**
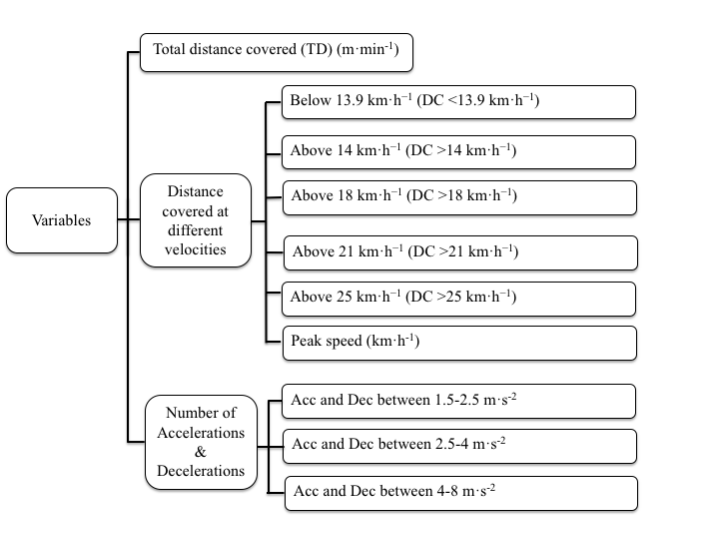
External load analyzed in official, friendly, training matches and modified-sided games.

The variables used to compare the external loads of matches and MSGs are presented in Figure 1. Since the analyzed tasks had different duration (official and friendly matches = 90 min and training and MSGs = 30 min), data were normalized by minutes to ensure correct analysis, as has been done in previous studies (Clemente et al., 2019a; Dalen et al., 2019; Martin-Garcia et al., 2019).

### Procedures

Time-motion analysis was conducted during competitive (official) and non-competitive matches (friendly, training and MSGs). During the first half of the season, 12 official matches, 7 friendly matches, 6 training matches and 3 MSGs were monitored. Official and training matches and 3 friendly matches were recorded in the same stadium, with a dimension of 100 x 60 m, while 4 friendly matches were played on fields with similar characteristics (length: 98.5 ± 0.71 m and width: 59.7 ± 1.5 m), maintaining a relative area per player of around 300 m^2^. While official and friendly matches had duration of 90 min (extra time and substituted players were excluded), training matches were played over a period of 30 min with the same official rules as friendly and official matches. At least 72 hours elapsed between each match, opposing teams were always of a similar level, and the match format was kept constant to reduce any variability in the players’ physical performance (Rampinini et al., 2007). The team used a 1–4–3–3 formation, comprising 2 central defenders, 2 fullbacks, 3 central midfielders and 3 strikers during all matches. The MSG was designed by 2 coaches with more than 10 years of professional experience, to achieve a specific scenario (simulating decision-making in matches) to reproduce the running demands of competition, but with fewer players and a shorter time. It was played with 5 *vs*. 5 + goalkeepers on half field dimensions (50 x 60 m) to maintain the same relative area per player as that achieved in actual matches (300 m^2^). This drill was used over periods of 30′ (6 x 5′ with 2′ of passive recovery). During rest periods, players could drink fluids *ad libitum*. All participants were advised to maintain their normal diet, with special emphasis being placed on high intake of water and carbohydrates (Casamichana et al., 2012). To ensure the continuity of matches, several balls were collocated around the zone of play. Likewise, to ensure maximal intensity and motivation during play, the coach always provided verbal encouragement (Rampinini et al., 2007).

### Statistical Analyses

Data are presented as mean ± standard deviation (SD). The normality of distribution of the variables was verified using the Shapiro–Wilk test. Differences in official, friendly, training and MSG matches were analyzed using one-way analysis of variance (ANOVA). A post hoc test was conducted with Bonferroni correction for multiple comparisons. In addition, possible differences or within-matches changes were analyzed for practical significance using magnitude-based inferences, pre-specifying 0.2 between-subject SDs as the smallest worthwhile effect (Hopkins et al., 2009). The standardized difference or effect size (ES, 90% confidence limit [90%CL]) in the selected variables was calculated. Threshold values for assessing the magnitude of the ES (changes as a fraction or multiple of baseline standard deviation) were <0.20, 0.20, 0.60, 1.2, and 2.0 for trivial, small, moderate, large, and very large, respectively (Hopkins et al., 2009). Quantitative chances of higher or lower changes were evaluated qualitatively as follows: <1%, almost certainly not; 1–5%, very unlikely; 5–25%, unlikely; 25–75%, possible; 75–95%, likely; 95– 99%, very likely; >99%, almost certain (Hopkins et al., 2009).

## Results

The running activity in official, friendly, and training matches and MSGs is presented in Table 2. Analysis of the data indicated that official matches produced statistically higher peak speeds than the other studied scenarios (very large magnitude). Likewise, in official matches, soccer players covered statistically more TD and DC <13.9 km·h^-1^ than in training matches (very large and large magnitude), but lower TD, DC <13.9 km·h^-1^ and DC >14 km·h^-1^ than in friendly matches (moderate magnitude). When the running activity between friendly and training matches was compared, it was found that friendly matches achieved greater TD, DC <13.9 km·h^-1^ and peak speed than training matches, but training matches produced more DC >25 km·h^-1^ (very large and large magnitude). In MSGs, soccer players covered statistically more TD, DC <13.9 km·h^-1^ and DC >14 km·h^-1^, but less DC >21 km·h^-1^ and DC > 25 km·h^-1^ than in official and friendly matches and less DC >25 km·h^-1^ than in training matches (very large to moderate magnitude).

**Table 2:** Running activity in official, friendly and training matches and modified-sided games.

	TD (m/min)	DC <13.9 (km/h)	DC >14 (km/h)	DC >18 (km/h)	DC >21 (km/h)	DC >25 (km/h)	Peak speed (km/h)
Official Matches	102.2 ± 5.5	81.3 ± 3.2	20.8 ± 3.6	8.8 ± 2.0	4.4 ± 1.1	1.3 ± 0.3	32.5 ± 0.9
Friendly Matches	107.4 ± 6.2	85.2 ± 4.6	22.1 ± 3.3	8.8 ± 2.1	4.0 ± 1.3	1.1 ± 0.5	29.4 ± 1.0
Training Matches	94.9 ± 12.2	76.3 ± 11.8	18.4 ± 4.1	7.8 ± 3.4	4.2 ± 2.7	1.5 ± 1.5	27.9 ± 2.2
MSG	116.1 ± 7.6	90.9 ± 7.3	24.9 ± 3.3	9.1 ± 2.3	3.6 ± 1.2	0.5 ± 0.3	27.1 ± 1.9
	
Difference in means (%;±90% CL)							
	
Official vs. Friendly	0.87 ± 1.38 Moderate	0.85 ± 1.38 Moderate	0.61 ± 0.57 Moderate	0.21 ± 0.69 Small	-0.17 ± 0.81 Small	-0.56 ± 1.12 Moderate	-1.40 ± 0.50 Very Large**
Official vs. Training	-1.68±1.46 Large	-2.40 ± 2.04 Very Large*	0.13 ± 0.94 Small	-0.10 ± 1.14 Small	-0.63 ± 1.57 Moderate	0.21 ± 2.40 Small	-2.05 ± 2.25 Very Large**
Official vs. MSG	2.21 ± 0.77 Very Large**	2.10 ± 0.80 Very Large**	1.07 ± 0.33 Moderate	0.0 ± 1.0 Small	-0.98 ± 0.32 Moderate	-2.53 ± 0.50 Very Large**	-2.20 ± 0.90 Very Large**
Friendly vs. Training	-2.19 ± 0.77 Very Large**	-2.22 ± 0.24 Very Large**	0.0 ± 0.21 Small	-0.04 ± 0.4 Small	-0.36 ± 0.55 Small	1.33 ± 0.77 Large	-1.34 ± 0.66 Large
Friendly vs. MSG	1.24 ± 0.87 Large	1.06 ± 0.84 Moderate	0.72 ± 0.58 Moderate	0.06 ± 0.77 Small	-0.51 ± 0.73 Moderate	2.13 ± 0.85 Very Large*	-1.60 ± 1.81 Very Large*
Training vs. MSG	1.52 ± 0.50 Large*	1.22 ± 0.39 Large*	0.92 ± 0.85 Moderate	0.24 ± 0.81 Small	-0.05 ± 0.78 Small	-0.92 ± 0.80 Moderate	-0.40 ± 0.9 Small

MSG = Modified-sided game; TD = Total distance; DC = Distance covered; CL = Confidence limits; * p < 0.05

** p < 0.01

The acceleration and deceleration demands of official, friendly, and training matches and MSGs are presented in Table 3. Players in MSGs performed a significantly greater number of accelerations and decelerations of all studied types than in matches (very large and large magnitude). Likewise, friendly matches achieved a significantly greater number of accelerations between 1.5–2.5 and 2.5–4 m·s^-2^, but fewer decelerations between 4–8 m·s^-2^ than official and training matches (very large to small magnitude) and performed significantly more decelerations between 1.5–2.5 m·s^-2^, but fewer accelerations between 2.5–4 m·s^-2^ than official games (moderate magnitude). In training matches, there were significantly more accelerations between 4–8 m·s^-2^ than in other studied matches (large magnitude).

**Table 3:** Practical implications.

	MD	MD +1	MD +2	MD -4	MD -3	MD -2	MD -1
Training week		Active recovery		Option 1			
		65′		70′ FM or TM (i.e.			
	70′	(stretching, mobility, UB, low intensity activity)	Day off	non-starter second team) 10′ PS exercise			
		Option 1					
		70′ FM or TM			10′ Warm-up	10′ Warm-up	10′ Warm-up
		(i.e. non-starter 2nd	Complementary		15′ TT SSG	10′ SSG	10′ TT activation
		team)	training	Option 2	ARP ≈80 m^2^	ARP <70 m^2^	10′ SSG
		10′ PS exercise	10′ Warm-up	10′ Warm-up	30′ TT exercise	20′ TT exercise	ARP <70 m^2^
			30′ Strength training	10′ TT activation	30′ TM	15′ Strategic	10′ TM
	<70′	Option 2	and mobility	10′ PS exercise			
		10′ Warm-up	20′ Analytic training	30′ MSG			
		10′ TT activation	(HS, VHS or S	ARP ≈300 m^2^			
		10′ PS exercise	exercise)	30′ TM			
		30′ MSG					
		ARP ≈300 m^2^					
		30′ TM					

Time		65′/ 80′ - 90′	0 / 60′	80′ - 90′	85′	65′	40′
Acc/Dec	***/ *	NR /***	NR /	***	**	**	*
HS/VHS	***/ *	NR / ***	NR /	***	**	*	**
S	***/ *	NR / ***	NR /	***	*	*	*

MD = match day; FM = friendly match; TM = training match; UB = upper body strength; PS = peak speed; TT = technical-tactical; MSG = modified-sided game; SSG = small-sided game; ARP = area relative per player; Acc/Dec = acceleration and deceleration demands; HS/VHS = distance covered >18 and 21 km·h-1; S = distance covered >25 km·h-1 and peak speed; NR = No requirements; *** = high demands; ** = moderate demands; * = low demands.

## Discussion

The purpose of this study was to compare external loads in competitive (official) and noncompetitive matches (friendly, training and MSG) in professional soccer players. The main findings of this study were: (1) official matches showed higher peak speeds than other studied scenarios; (2) official, friendly, and training matches had similar requirements at higher speeds among them, and higher than MSGs, but less total distance covered and lower speeds than MSGs, and (3); there were a greater number of accelerations and decelerations in training exercises than in matches, in all studied ranges.

In soccer, it is accepted that match status influences teams’ behavior (Taylor et al., 2008) and that it is more significant in official competition, in which the result is obviously more important both for the team and for each player. This may explain why our players achieved the highest peak speeds in official games, compared with the rest of scenarios, even though they had the same relative area per player. Although this finding is in line with the previously published data (Casamichana et al., 2012), it is remarkable that the magnitude of peak speed seems quite higher. Casamichana et al. (2012) reported a lower peak speed in friendly matches (27 ± 1.8 km·h^-1^) than the one in the present study (32.5 ± 0.9 and 29.4 ± 1.0 km·h^-1^, official and friendly matches, respectively), but similar to our training matches and MSGs (27.9 ± 2.2 and 27.1 ± 1.9 km·h^-1^, respectively). Despite that previous investigations have established that peak speed is related to available space in SSGs, being higher when the area is greater (Castellano and Casamichana, 2013; Hill-Haas et al., 2009; Lacome et al., 2018), these peak speed differences cannot be explained by the matches and MSGs size, since both studies used the same relative area per player (300 m^2^). There are numerous reasons which could partly influence this variable, like a playing formation (1-4-4-1-1 vs. 1-4-3-3), the competitive level (semiprofessional vs. professional), the Ligue in which teams compete, etc. Thus, these differences cannot be explained solely by our study, and it strongly suggests the need of more studies to clarify the influence of those possible determinants.

To our knowledge, this is the first study comparing the running activity of official, friendly, and training matches maintaining the same characteristics (11 *vs*. 11, rules, and same playing area). Except a large difference in DC >25 km·h^-1^ between friendly and training matches, all differences in moderate to high intensity running between the different scenarios (DC >14 km·h^-1^, DC >18 km·h^-1^, DC >21 km·h^-1^, and DC >25 km·h^-1^) were small or moderate. The main finding of the present study shows that the combination of friendly and training matches could be a suitable way to prepare soccer players for official competition because this combination can almost reproduce all the moderate and high intensity running requirements of official matches (except peak speed, as previously noted). To our knowledge, only one study has compared running demands between official and training matches (but not friendly matches) reproducing most of the demands of competition (Martin-Garcia et al., 2019). However, that study described an over-stimulation of the sprint demands, in comparison with our study, probably related to the duration of the sessions (10 vs. 30 minutes, respectively), since increasing the time of execution of a training task is associated with a diminution in intensity (Fanchini et al., 2011). From a practical application point of view, we think that this preliminary analysis performed by our team should be replicated (always maintaining all characteristics described) to confirm these results.

Comparing matches with MSGs, our outcomes are in line with most of those previously published, describing a greater running activity at higher intensity in matches than in small-, medium- or large-sided games (Casamichana et al., 2012; Clemente et al., 2019a; Gómez-Carmona et al., 2018; Martin-Garcia et al., 2019). Only one study found greater running activity at high speed and while sprinting in a 4 *vs*. 4 + 4 floaters SSGs than in matches (Dellal et al., 2012).

**Figure 2:**
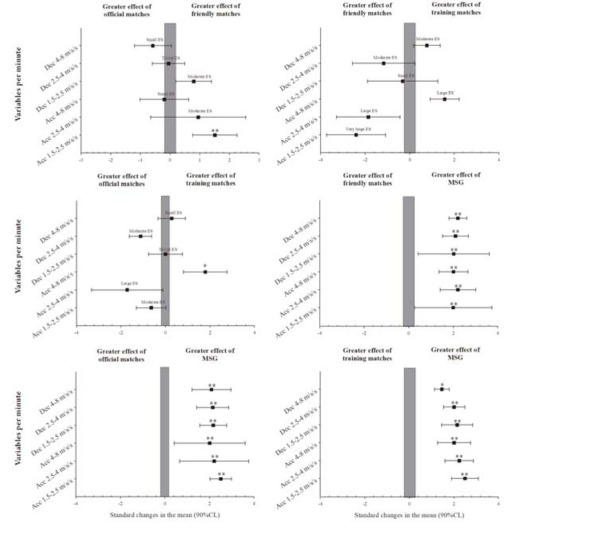
Acceleration and deceleration demands of official, friendly, training matches and MSGs. MSG = modified-sided game; Acc = acceleration; Dec = deceleration; CL = Confidence limits; * p < 0.05 **p < 0.01

Is seems that the numerical superiority generated with the floaters in that study could have caused this difference. Previous studies have confirmed that in SSGs the distances covered at high speed and while sprinting are increased when the relative area per player is higher (Clemente et al., 2019a, 2019b; Gaudino et al., 2014; Sarmento et al., 2018). Although our MSGs provided 300 m^2^ per player, their running activities above 21 and 25 km·h^-1^ were lower. For this reason, it appears that the relative area per player alone is not sufficient to increase running activity at these intensities and there must be an appropriate balance between the absolute area and the number of players.

In soccer, matches and training have multidimensional demands and the use of only a single intensity training load may lead to an underestimation of physical demands (Casamichana et al., 2019). Thus, in addition to the distance covered at different speeds, the number of accelerations and decelerations should be studied when comparing the running demands of this sport. Our results showed that MSGs had more accelerations and decelerations in all studied ranges than other matches. These data are in line with the published literature, which confirms that SSGs have greater requirements for changes of speed and direction than friendly matches (Castellano and Casamichana, 2013) and official matches (Clemente et al., 2019a; Dalen et al., 2019; Gómez-Carmona et al., 2018; Martin-Garcia et al., 2019). In comparison with friendly matches, the literature has indicated that SSGs (with a 210 m^2^ area per player) achieved a greater number of accelerations between 1.0-1.5, 1.5-2.0 and 2.0-2.5 m·s^-2^, but not >2.5 m·s^-2^ than matches for semi-professional players (Castellano and Casamichana, 2013). In professional players, some authors showed that a 5 *vs*. 5 + 2 goalkeepers’ format (a 132 m^2^ relative area per player) produced ~ 150% difference with respect to the most demanding sections of the match (Martin-Garcia et al., 2019) and that the average number of accelerations per minute was 113% higher in 4 *vs*. 4 + 2 goalkeepers (a 190 m^2^ relative area per player) and 49% higher in 6 *vs*. 6 (a 184 m^2^ relative area per player) formats than in official matches (Dalen et al., 2019). Gómez-Carmona et al. (2018) also found a lower number of changes of speed demands in official matches than in 6 *vs*. 6 with a lower relative area per player (83 m^2^) in professional players. Similarly, Clemente et al. (2019b) reported a greater number of accelerations in 5 *vs*. 5 + 2 goalkeepers (a 124 m^2^ relative area per player ) than in 6 *vs*. 6 + 2 goalkeepers, 9 *vs*. 9 + 2 goalkeepers formats and official matches (with a 120, 194 and 260 m^2^ relative area per player, respectively). All these studies used lower relative areas per player in their training tasks than in matches, confirming that a lower relative area per player increases the number of accelerations and decelerations. In contrast, our MSGs (with the same area per player as in official matches) showed a greater number of changes of speed, indicating that a lower number of players can increase the number of accelerations and decelerations. It is known that a smaller number of players results in more duels, dribbles, and ball touches (Dellal et al., 2012; Owen et al., 2011, 2014; Sarmento et al., 2018), thus to deal with these situations, players perform more accelerations and decelerations.

Only small differences in changes of speed were found between official, friendly, and training matches with the same demands. Thus, although SSGs are clearly useful to stimulate accelerations and decelerations in soccer (Clemente et al., 2019a; Dalen et al., 2019; Gómez-Carmona et al., 2018; Martin-Garcia et al., 2019), the combined use of friendly and training matches could be, at least partly, an adequate solution to address the differences from official matches. Although coaches should consider acceleration and deceleration demands because they carry a significant mechanical load and can increase fatigue in soccer players (Gaudino et al., 2014), only one study compared accelerations and decelerations (>3 m·s^-2^) in training and official matches, finding higher demands in training matches (Martin-Garcia et al., 2019).

Although this study provides new insights in understanding the external load of different match scenarios for soccer players, it does have some limitations. Firstly, only one MSG format of 5 *vs*. 5 + 2 was used, thus these data cannot be extrapolated to MSGs with more or fewer players. Secondly, this study did not assess running activity according to a playing position so it is not known whether the running behavior of soccer players in these matches could be affected by their playing position. For these reasons, futures studies should compare official, friendly and training matches with MSGs with different numbers of players, but a 300 m^2^ relative area per player, analyzing running demands according to the playing position.

## Conclusions

The results of the present study demonstrated that official, friendly, and training matches and MSGs had a particular external load profile. In summary:

• In official matches soccer players achieved higher peak speeds than in any other match scenario (friendly and training matches or MSGs).

• Except a large difference in DC >25 km·h^-1^ between friendly and training matches, all differences in moderate to high intensity running (DC >14 km·h^-1^, DC >18 km·h^-1^, DC >21 km·h^-1^, and DC >25 km·h^-1^) between official, friendly, and training matches were small or moderate. This suggests that a combination of friendly and training matches could be a suitable means to prepare soccer players for the running requirements of official competition.

• Soccer players in MSGs exhibited more accelerations and decelerations than in official, friendly, and training matches.

## Practical Implications

Understanding that there is clearly a challenge to loading players in a soccer-specific context, especially with high speed running (Akenhead et al., 2016), and that it may be necessary to include varied types of tasks to overload the player during the training process (Martin-Garcia et al., 2019), this study can help soccer coaches prepare their training schedules (Table 3) to optimize performance of their players. On the one hand, this work demonstrates that friendly and training matches, in conjunction with MSGs, are suitable tools to prepare players for official matches because each of them provides training in a specific area, although for complete preparation training should include tasks which simulate the peak speed of official matches. On the other hand, to overload changes of speed with respect to official matches, coaches can use the suggested MSG, but if circumstances allow, the addition of friendly and training matches should be considered to address the accelerations and decelerations encountered in official competitions.
